# Developing a Conceptual Framework of Seroadaptive Behaviors in HIV-Diagnosed Men Who Have Sex With Men

**DOI:** 10.1093/infdis/jiu482

**Published:** 2014-12-01

**Authors:** Minttu Rönn, Peter J. White, Gwenda Hughes, Helen Ward

**Affiliations:** 1 Department of Infectious Disease Epidemiology; 2 MRC Centre for Outbreak Analysis and Modelling and NIHR Health Protection Research Unit in Modelling Methodology, Department of Infectious Disease Epidemiology, School of Public Health, Imperial College London; 3 Modelling and Economics Unit, Centre for Infectious Disease Surveillance and Control; 4 STI Section, Centre for Infectious Disease Surveillance and Control, Public Health England, London, United Kingdom

**Keywords:** HIV, men who have sex with men, seroadaptive

## Abstract

***Background.*** Seroadaptive behaviors are strategies employed by men who have sex with men (MSM) to reduce the transmission risk for human immunodeficiency virus (HIV). It has been suggested that they contribute to the increasing diagnoses of sexually transmitted infections in HIV-diagnosed MSM. To understand the context in which the reemerging sexually transmitted infections appear, we developed a social epidemiological model incorporating the multiple factors influencing seroadaptive behaviors.

***Methods.*** A literature review of seroadaptive behaviors in HIV-diagnosed MSM was conducted. The literature was synthesized using a social epidemiological perspective.

***Results.*** Seroadaptive behaviors are adopted by MSM in high-income countries and are a way for HIV-diagnosed men to manage and enjoy their sexual lives. Influences are apparent at structural, community, interpersonal, and intrapersonal levels. There is little evidence of whether and when the behavior forms part of a premeditated strategy; it seems dependent on the social context and on time since HIV diagnosis. Social rules of HIV disclosure and perception of risk depend on the setting where partners are encountered.

***Conclusions.*** Seroadaptive behaviors are strongly context dependent and can reduce or increase transmission risk for different infectious diseases. Further data collection and mathematical modeling can help us explore the specific conditions in more detail.

Men who have sex with men (MSM) have been disproportionately affected by the human immunodeficiency virus (HIV) epidemic [1]. The increasing life expectancy of HIV-positive individuals results in increasing prevalence of HIV among MSM, which has an impact on social and sexual norms [2, 3] and a resulting impact on rates of other sexually transmitted infections (STIs). The rate of STIs fell in the 1980s and early 1990s after behavioral change in response to HIV but this then reversed; for example, gonorrhea diagnoses in MSM increased from late 1990s onward in the United Kingdom [4], followed by outbreaks of syphilis, sexually acquired hepatitis C, and lymphogranuloma venereum, which have particularly affected HIV-positive men [5]. The reemergence of STIs has been attributed to treatment optimism (leading to declining concern about HIV), safer sex fatigue, and seroadaptive behaviors [2, 3].

Seroadaptive behaviors have been seen as a harm reduction strategy and a functional response to the HIV epidemic [6–8]. The term refers to any modification of sexual behavior based on the person's (perceived) serostatus, the (perceived) status of the partner and/or HIV transmission risk by type of sex act [9, 10]. On a practical level, seroadaptivity alters mixing patterns in the population with increased like-with-like mixing by serostatus (serosorting) or, in a serodiscordant partnership, trying to reduce probability of HIV transmission by having the HIV-negative partner take the insertive position in anal sex (strategic positioning) or trying to limit exposure to the virus (such as by performing withdrawal before ejaculation, using viral load as a measurement of transmission risk, or refraining from anal sex) [11].

The consequences of seroadaptive behaviors depend on HIV status. For HIV-negative men, they can reduce the risk of HIV acquisition, but this is dependent on levels and frequency of HIV testing [12], the prevalence of undiagnosed HIV infection [13], and full disclosure by all parties. For the HIV-negative person, it remains a high-risk strategy in most settings, superseded only by not having any risk management strategy at all [14]. For HIV-diagnosed men, seroadaptive behaviors are a way to limit onward transmission and to manage one's sexual identity in the presence of HIV infection. Therefore, disclosure of HIV status and protected sex is no longer about protecting oneself but it can be seen as a moral responsibility and an altruistic act toward others. The main personal risks center on acquisition of other STIs.

The overall aim of our review was to examine factors that influence seroadaptive behaviors. There are no agreed-on definitions of seroadaptive behaviors, and we tried to determine how the behaviors are understood in the scientific literature and the pathways that facilitate the behaviors. Even when seroadaptive behaviors are “successfully” practiced (reducing the risk of HIV transmission), STI transmission risk may still remain. Hence, understanding the determinants of seroadaptive behaviors can help us understand how they contribute to increasing rates of STIs. The framework can also act as a platform for hypothesis generation in statistical analyses of behavioral data, inform mathematical modeling, identify gaps in our knowledge, and aid in planning interventions.

## METHODS

We performed a literature review using the following search words and Boolean operators: serosorting OR seroadaptive OR “strategic positioning” OR “sexual harm reduction” OR serodisclosure OR (serostatus AND disclosure) OR “negotiated safety” OR serodiscordan* OR seroconcordan* OR (“HIV status” AND partner) AND (“men who have sex with men” OR homosexual*). PubMed and Web of Knowledge were used as the search platforms. The literature search is described in more detail in the supplementary material.

Four categories were predetermined as levels of the framework: structural, community-level, interpersonal, and intrapersonal factors, adapted from the approach of the social epidemiological framework for HIV proposed by Poundstone et al [15], which was used to identify mechanisms through which behaviors at the population level may be influenced. The social resistance framework offered by Factor et al [16] was also used to guide the analysis. Factor et al both complement and challenge the social epidemiology approach of Poundstone et al by describing structural inequalities while acknowledging the role of individual agency. In the framework of Factor et al [16], health disparities are explained by detachment of the nondominant group from the dominant group's culture; the collective identity of the nondominant group results in opposition against the (health) values created by the dominant group. This enables us to contrast the individual agency of an HIV-diagnosed individual and the wider determinants for his or her health and behavior. In the narrative synthesis, we focused on studies in which seroadaptive behaviors were a main component and those focusing on HIV-diagnosed men and their partnerships.

## RESULTS

**Figure 1.**
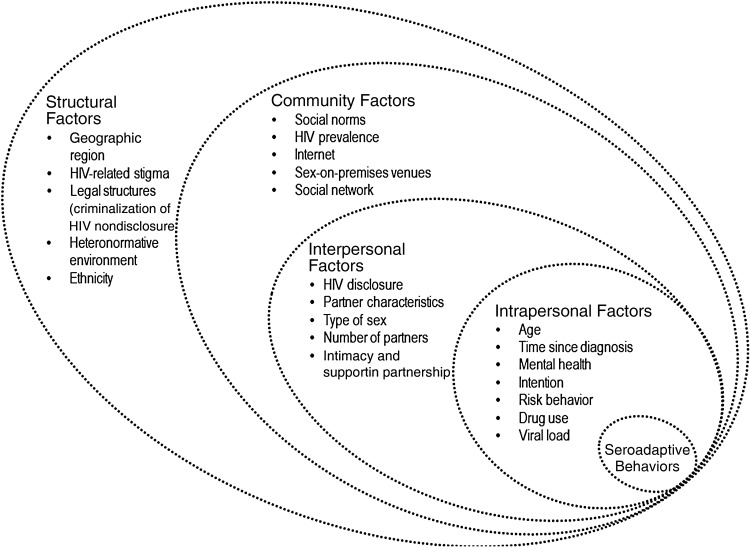
Conceptual framework of the social epidemiology of seroadaptive behaviors in human immunodeficiency virus (HIV)-diagnosed men who have sex with men.

The search resulted in 633 publications, of which 199 of the most relevant articles were reviewed for this study. In this article, we present a summary of the findings; the full review is presented as part of a PhD thesis [17]. A number of prerequisites are needed for seroadaptive behaviors to occur: awareness of HIV transmission risks, availability and uptake of HIV testing, disclosure of HIV status, sufficient prevalence of HIV in the community, and HIV-related attitudes that facilitate seroadaptive behaviors. A conceptual framework is presented in Figure 1, and the factors presented in the framework are summarized in Table 1 and discussed in the next section.

**Table 1.** Key Components in the Conceptual Framework of Seroadaptive Behaviors in HIV-Diagnosed MSM

Factors	Connection to SABs in HIV-Positive MSM
Structural factors	
Geographic region	Most evidence of SABs originates from high-income countries.
Criminalization of HIV	Behavior may be altered owing to concern over prosecution.
Social inequalities	HIV-related stigma, heteronormative environment, and ethnicity can affect disclosure of HIV status
Community-level factors	
Social norms	Norms can either support or marginalize the HIV-positive population, depending on setting; social and sexual networks are likely to overlap, and attitudes in a social network are likely to influence sexual behaviors; SABs also require a sufficient pool of HIV-diagnosed individuals, which may affect formation of HIV-positive subcultures
Internet	Can facilitate SABs through specialized Web sites
Sex-on-premises venues	Unless the venue/event is for HIV-positive individuals, increased anonymity makes consistent SABs less likely
Interpersonal factors	
HIV disclosure	Necessary precursor for informed SABs; seroconcordancy is associated with UAI; HIV disclosure (verbal or nonverbal) is considered a key component of SABs
Partner characteristics	SABs were more consistent in long-term than in short-term partnerships.
Type of sex	Strategic positioning may be practiced among HIV-positive MSM; lack of disclosure may be managed by choosing less risky types of sex
No. of partners	Increased anonymity and decreased responsibility are associated with number of partners, making SABs less likely
Intimacy and support	In long-term partnerships, UAI may be favored in place of protected sex; concordant partnerships may increase the sense of intimacy.
Intrapersonal factors	
Time since diagnosis and age	SABs may be time dependent.
Intention and self-efficacy	Likely to be an important determinant for consistent SABs and practicing safer sex
Risk behavior	Related to various other factors, probably making consistent SABs less likely
Drug use	Increased overall risk behavior is associated with increased drug use
Viral load	It is unclear how widely viral load is used as a SAB

Abbreviations: HIV, human immunodeficiency virsus; MSM, men who have sex with men; SAB, seroadaptive behavior; UAI, unprotected anal intercourse.

There is observational evidence that serosorting contributes to the increasing trends of STIs in HIV-diagnosed MSM. In Germany, HIV-diagnosed men who reported either strategic serosorting (preferred partners of the same status) or tactical serosorting (using a condom if a partner is not the same serostatus or is of unknown status) were more likely to have had a bacterial STI within the past 12 months than nonserosorting HIV-diagnosed men. Neither form of serosorting was associated with bacterial STIs among HIV-negative men [18]. In San Francisco, the leveling off of HIV incidence at the same time as an increase in syphilis and gonorrhea diagnoses was suggested to be due to an increase in serosorting [19].

### Structural Factors

#### Geographic Region and Culture

Most studies identified in this review were from high-income Western countries. Little evidence exists outside this area. In an online survey conducted in Asia [20], there was some variation in disclosure patterns for HIV-diagnosed MSM between countries; Chinese men were the least likely to disclose (88% were nondisclosers), with the fewest nondisclosers among Philippine men (53%), although sample size per country was relatively small. A partner's nondisclosure was the strongest explanatory factor for a respondent's nondisclosure in the multivariable model. The authors interpret this to mean that serosorting is less common in Asian populations, attributing the difference to stigma and discrimination. Disclosure is also rare in Bangkok, Thailand, with 61% of HIV-diagnosed MSM reporting no disclosure to their last sexual partner and with most men having protected sex, although those with an HIV-positive partner reported less protected sex than those with HIV-negative or unknown-status partners (61%, 85%, and 91%, respectively) [21]. In another Bangkok-based study, the HIV status of the steady male partner of an HIV-infected MSM was not significantly associated with having reported unprotected sex in the past 3 months; an association was present only for having a steady male partner [22].

#### Criminalization of HIV

Criminalization of a behavior is an extreme form of state control, and its application to HIV transmission might influence how HIV-diagnosed individuals choose their partners. In a qualitative study of HIV-diagnosed men in Seattle and Los Angeles, men who always disclosed their status expressed fear over legal consequences as a reason for disclosure [23]. However, a qualitative research study in England and Wales demonstrated misconceptions as to which types of situations could be prosecuted [24].

#### Social Inequalities

A meta-analysis explored the variation in risk for HIV infection for black MSM in North America and the United Kingdom [25]. In a subanalysis for HIV-diagnosed men, black men were less likely to disclose their HIV status to partners than other HIV-diagnosed MSM (summary estimate from 3 studies: odds ratio [OR], 0.5; 95% confidence interval, .3, .8). They were not significantly different in their reporting of serodiscordant unprotected anal intercourse (sdUAI), serosorting, or strategic positioning, and there were several inequalities in HIV care between the 2 groups. A theoretical model validated with data found that among HIV-diagnosed MSM, internalized heterosexism (negative attitudes toward homosexuality) was indirectly associated with greater HIV transmission risk (unprotected anal intercourse [UAI] with an HIV-negative or unknown-status partner) and reduced adherence to antiretroviral therapy (ART) [26]. Internalised heterosexism was further associated with negative affect (negative emotional states), stimulant use and poorer ART adherence in the statistical analyses.

### Community-Level Factors

#### Gay Community Social Norms

It has been suggested that HIV testing has increased marginalization of HIV-diagnosed men; early on in the HIV epidemic a shared accountability for HIV transmission was advocated by assuming everyone to be infected. With HIV testing the community risk transformed into an individual- or partner-level risk, and a “hierarchy of risk” emerged with groups at risk (HIV negative), groups potentially presenting a risk (HIV untested) and groups presenting a risk (HIV-diagnosed) [27]. Observational data indicate that social norms about condom use, social support, and perceived control over sexual behavior mediate intentions for safer sex, which in turn have an inverse association with sdUAI [28]. Scotland, an area with low HIV prevalence, was described as having a “universal HIV-negativity assumption” and the social cost of being HIV-diagnosed was given as one reason not to get tested [27]. In contrast, HIV positivity can also become the norm, as described in a qualitative study from San Francisco, where HIV-negative men described a feeling of exclusion and a sense of fatalism about HIV acquisition [29].

#### Role of the Internet

The Internet can facilitate disclosure of HIV status before partnership formation but also increase sexual mixing between high-risk populations. A French study recruited almost 14 000 MSM using gay Web sites; 63% of HIV-diagnosed men entered the study via a specialized gay Web site (specializing in unprotected and fetish sex), compared with 32% of HIV-negative men. A wider range of sexual practices and higher sexual activity levels were reported by the HIV-diagnosed men, and they had more UAI with partners of known or unknown serostatus [30]. In another French study, HIV-diagnosed men who reported serosorting with a casual partner were more likely to look for sex partners online and less likely to look for partners in cruising venues than HIV-diagnosed men who reported UAI with a casual partner but not serosorting [31]. Among MSM from Toronto, the participants talked about “unspoken knowledge,” whereby formal disclosure is mediated via indirect messages, such as not providing HIV status or stating “safe sex only” on an Internet profile to indicate that the person is HIV positive [32].

#### Venue Type

In some locations, with studies mainly arising from North America, public sex areas have been described as the territory of HIV-diagnosed men with public sex “rules” of no disclosure and no condoms [33]. However, the type of venue and social norms of the setting are likely to play a role. The rates of sdUAI vary between venues: in California sdUAI was most commonly reported among MSM attending circuit parties (30%), followed by those in cruising areas (16%), and it was least commonly reported by those who did not visit a gay venue (4%) [34]. “positive (POZ) parties” were specifically created as events to facilitate sex between HIV-diagnosed MSM. The most important reasons stated for attending a POZ party were freedom from HIV disclosure, having uninhibited or unrestricted sex, and not having to worry about infecting others [35].

### Interpersonal and Intrapersonal Factors

The concept of seroadaptive behavior strategies arose some time into the HIV epidemic, but relevant practices were reported early on; in a sample of HIV-diagnosed MSM from Los Angeles in 1991 it was noted that though <10% reported unprotected sex, it was more likely to occur with other HIV-diagnosed men [36]. Seroconcordant UAI (serosorting) among HIV-diagnosed men has been associated with personal beliefs about the consequences of unprotected sex, such as risk perception of STIs and perceived responsibility over partner's health, but also with hedonistic expectations about sex and drug use (particularly with methamphetamine) [37].

#### Partners, Partnerships, and HIV Disclosure

Disclosure can be seen by some men as transferring the responsibility (of risk) to the partner, and it can lead to UAI in a seroconcordant partnership [38]. In more anonymous settings, disclosure is mitigated by perceived serostatus based on circumstantial evidence and normative assumptions based on the setting of the encounter [39].

Disclosure of HIV status, seroconcordance, and the type of relationship between partners create a context where unprotected sex can occur, and seroconcordance can be a predictor of UAI, possibly based on a decision-making process between partners [40]. Similar findings have been reported elsewhere, with responsibility to the partner, knowing the partner, and having sought health information earlier were predictors of HIV disclosure [23, 41, 42]. HIV status was important but not the most important factor in men's perceptions of successful partnerships. Intimacy in partnership can increase condom use, but both seroconcordant and serodiscordant couples also list intimacy as a reason for UAI [43, 44].

The intentional practice of seroadaptive behaviors has received relatively little focus. In a longitudinal study of seroadaptive behaviors from San Francisco [45], strategic positioning was the intention sexually active HIV-diagnosed men were most likely to adhere to during 12-month follow-up (always receptive; 41% of those with this intention reported adhering to it), whereas 28% practiced pure serosorting. In a Swiss study [46], only 8% of HIV-diagnosed men (who had had UAI with casual male partners in the prior 12 months) reported strategic positioning, whereas serosorting was reported by 41% and withdrawal before ejaculation by 33%.

#### Cohort Effects: Time Since HIV Diagnosis and Age

Few longitudinal studies have explored the changing risk profile after HIV diagnosis. A longitudinal study from San Francisco recruited MSM during early HIV infection and followed them up over time [9]. Marked changes in both partner numbers and sdUAI were reported, with a mean of 4.2 (95% confidence interval, 2.7–6.6) potentially sdUAI partners 3 months before diagnosis, reduced to 0.9 (.5–1.7) by 12 months after diagnosis, followed by a slight increase to 1.7 (.9–3.1) at 48 months. Another study (using series of cross-sectional data) found a more complicated pattern of associations, with the biggest change in sexual behavior during the first year after diagnosis. Longer time since HIV diagnosis was associated with sdUAI, including with adjustment for age, but ceased to be significant when substance use and being on ART were added to the statistical model [47]. Time since HIV diagnosis and age are correlated, and younger age is associated with increased risk taking [48].

#### Well-Being and Mental Health

The HIV-diagnosed men labeled as inconsistent disclosers reported more high-risk behaviors than nondisclosers or consistent disclosers. Self-efficacy, intention, and connection to other HIV-diagnosed men were associated with being a consistent discloser, and it was suggested that inconsistent disclosers lacked strategies to manage their sexual risk taking, whereas nondisclosers may use alternative ways to avoid high-risk sex [49]. Among HIV-diagnosed MSM from the United States taking part in a qualitative study, health priorities included maintaining good mental health, help with substance abuse, and advice on stress coping mechanisms, and participants did not perceive health promotion programs focusing solely on safer sex as effective. Participants hoped for a support group that would focus on life management with a more holistic approach [50].

#### High-Risk-Behavior

The concept of intentional unprotected sex among MSM incurring HIV transmission risk (“barebacking”) has become more common since the early 2000s [51]. To HIV-diagnosed men who are “consistently barebacking,” promoting serosorting or strategic positioning might be a beneficial risk reduction approach [52]. However, the evidence is currently conflicting. In one intervention, conducted in 4 American cities, the intervention arm—receiving counseling on coping strategies, health, sexual health behaviors (such as disclosure), and negotiating safer sex—reported more serosorting at follow-up than the control arm [53]. Another peer-led behavioral intervention for safer sex found no statistically significant impact among HIV-diagnosed men, although the authors suspected this was due to a reverse impact on safer sex attitudes in some lower-risk participants who were exposed to ideas of risky sex by their peers. Focusing on self-protection instead of partner protection might also have more impact [54].

#### Drug Use

Party drugs and alcohol increase risk behavior, but one study indicated that for HIV-diagnosed MSM this may occur more with HIV-positive than with HIV-negative partners [55]. Methamphetamine use has been consistently shown to increase risk behavior, and HIV-diagnosed men also report more methamphetamine use than HIV-negative men [37, 56–58]. Among HIV-diagnosed substance-using MSM, those who had high coping self-efficacy (level of confidence of coping under stressful situations), positive coping behaviors, and less cognitive escape (use of substances during sex to escape from behavioural norms related to HIV transmission risk) were more likely to be consistently serosorting (defined as concordant partnerships) [6].

#### Viral Load

There is conflicting evidence for the use of viral load as a seroadaptive tool. In a meta-analysis of the prevalence of UAI among HIV-diagnosed MSM [59] receiving ART, high ART adherence or an undetectable viral load were not significantly associated with having engaged in UAI. In Sydney, Australia, undetectable viral load and optimism about ART were found to be positively associated with UAI among serodiscordant couples [60], and another Australian study [61] found that men who sometimes have UAI with casual partners were more likely than men who never do to report optimism about HIV treatment and use of sildenafil. In Chicago, Illinois, undetectable viral load was not associated with UAI; rather, risk was associated with optimism about HIV treatment [62].

## DISCUSSION

We have proposed a conceptual framework of seroadaptive behaviors in HIV-diagnosed MSM based on the published literature. Seroadaptive behaviors seem to be a phenomenon limited to high-income countries and not widely reported elsewhere. Seroadaptive behaviors are employed on partnership level with regular partners. When the anonymity of a sexual encounter increases (or when the number of partners increases), the perceived responsibility to disclose and discuss sex wanes, with more reliance on settings to infer risks. An example of this is the most high-risk settings, such as bathhouses and sex parties, where disclosure is seen as nonessential and where a separate microculture prevails.

The etiology of seroadaptive behaviors is not obvious based on studies conducted thus far, and the cross-sectional nature of many of the studies further limits causal inference. Seeking seroconcordant partnerships can be a conscientious risk management strategy, but social support and shared experiences gained from an HIV-diagnosed partner can be a significant factor in driving HIV-diagnosed men into a “seroadaptive environment” rather than avoidance of HIV transmission. Seroadaptive behaviors can also be a way of regaining some of the pre-HIV era freedom to have unprotected sex without fear of HIV transmission.

Narrative reviews are subjective by definition. The approach used here focused on the experience of HIV-diagnosed MSM, with the aim of understanding reasons and situations where seroadaptive behaviors occur. The strength of this review is that it looks at the variety of behaviors, which rarely occur in isolation, and it is thus able to capture some of the problems in the studies and the diversity of experiences. The framework is conceptual and has not been validated. Most published studies focused on high-risk MSM; men who consistently practice safer sex strategies or who are not sexually active are underrepresented.

The current review showed a pattern of seroadaptive behaviors that increase the like-with-like sexual mixing by HIV-diagnosed men. Given the high levels of unprotected sex, this can offer a pathway through which STIs have increased and—in the case of lymphogranuloma venereum, for example—reemerged in the HIV-diagnosed sexually active population. Assortative mixing between HIV-diagnosed MSM decreases the onward transmission of HIV and could be a viable risk-management strategy in some settings. However, many HIV-diagnosed men, especially in sex-on-premises venues, also report sdUAI.

Further studies looking at seroadaptive behaviors should better assess intention and whether and how seroadaptive behaviors are employed as a strategy. This would enable better estimation of how seroadaptive changes are affecting HIV and STI transmission through statistical and mathematical modeling as well as planning of health promotion programs for MSM. The biggest intervention challenge is to counteract forces of peer norms and drug abuse in small subcommunities in which traditional prevention messages are ineffective. From the qualitative work among HIV-diagnosed men, some expressed a need for programs that would focus on life management and not solely on sexual health. Given the diversity of experiences among HIV-diagnosed men, it is important to consider the dynamics of the specific community so as to adjust the message to the prevailing norm.
